# Natural Killer Cell Plasticity in Epithelial Ovarian Cancer and Their Therapeutic Implications

**DOI:** 10.3390/cells15141243

**Published:** 2026-07-09

**Authors:** Toshimichi Onuma, Meshach Asare-Werehene, Makoto Orisaka, Benjamin K. Tsang

**Affiliations:** 1 Department of Obstetrics and Gynecology, Faculty of Medical Sciences, University of Fukui, Fukui 910-1193, Japan; toonuma@u-fukui.ac.jp (T.O.); orisaka@u-fukui.ac.jp (M.O.); 2 Inflammation and Chronic Disease Program, Ottawa Hospital Research Institute, Ottawa, ON K1H 8L6, Canada; meshach.asarewerehene@lifelabs.com; 3 Lifelabs Medical Laboratory Services, Toronto, ON M9W 6J6, Canada; 4 Department of Obstetrics & Gynecology, Faculty of Medicine & Interdisciplinary School of Health Sciences, Faculty of Health Sciences, University of Ottawa, Ottawa, ON K1H 8L1, Canada; 5 Department of Cellular and Molecular Medicine & The Centre for Infection, Immunity and Inflammation (CI3), Faculty of Medicine, University of Ottawa, Ottawa, ON K1H 8M5, Canada

**Keywords:** natural killer cells, epithelial ovarian cancer, ascites, tumor microenvironment, immune suppression, compartment-specific immunity, NK cell dysfunction, NK cell therapy

## Abstract

**Highlights:**

- Natural killer (NK) cell dysfunction in epithelial ovarian cancer differs across peripheral blood, ascites, primary tumors, and metastatic lesions.
- Multiple disease site (peripheral blood, ascites, primary tumors, and metastatic lesions) assessment of NK provides a better prognostic index compared with only a single disease site.
- NK cell-targeted therapy should be tailored to the dominant disease site, with cytokine activation or intraperitoneal delivery for ascites-dominant disease and checkpoint-based or engineered NK cell strategies for primary or metastatic tumor-dominant disease.

**Abstract:**

Natural killer (NK) cells are key mediators of antitumor immunity; however, NK cell dysfunction in epithelial ovarian cancer should be considered not as a uniform defect, but rather as compartment-specific states that differ across the blood, ascites, primary tumor, and metastatic sites according to their local cellular interactions, soluble factors, and metabolic constraints. Peripheral blood provides an accessible systemic reference and may support immune monitoring. However, it does not fully reflect NK cell states in local or distant disease compartments. In ascites, cytokine-responsive and partially recoverable NK cell populations coexist with soluble, biochemical, and metabolic suppressive signals. In primary tumors, NK cells often acquire tissue-adapted suppressive phenotypes, characterized by altered activating receptors, increased inhibitory checkpoints, and reduced cytotoxic effector function. In metastatic lesions, NK cells appear to share suppressive phenotypes with primary tumors, although these phenotypes may be reinforced within metastatic niches through coordinated inhibitory receptor–ligand interactions. The above compartment-specific states imply that NK cell-targeted therapy for ovarian cancer should not rely on a unilateral strategy. Instead, therapeutic design may need to be multifaceted but coordinated, combining cytokine-based activation, adoptive NK cell transfer, checkpoint blockade, local delivery, and antigen-directed chimeric antigen receptor NK cell approaches according to the dominant biology of each compartment. Paired multi-compartment profiling and longitudinal functional assessment will be essential for biomarker development and compartment-guided treatment design.

## 1. Introduction

Advanced ovarian cancer (OVCA), which is often accompanied by malignant ascites and chemotherapy resistance, remains difficult to treat [1,2,3,4,5]. To improve outcomes in patients with poor prognosis, extensive research studies have focused on the tumor microenvironment, particularly, tumor immunity. Immune checkpoint inhibitors (ICIs) have markedly improved clinical outcomes in several malignancies, including melanoma, non-small-cell lung cancer, and renal cell carcinoma, and have become a central strategy in cancer immunotherapy [6,7,8]. These advances have also reinforced the importance of immune mechanisms in OVCA and have stimulated growing interest in the role of tumor immunity in disease progression and treatment resistance.

Natural killer (NK) cells are a key component of tumor immunity and possess intrinsic antitumor activity. NK cells are lymphocytes of the innate immune system that can recognize and kill tumor cells and virus-infected cells without prior antigen-specific sensitization. Their antitumor effects are generally mediated through the release of cytotoxic granules containing perforin and granzymes, expression of death receptor ligands, and production of cytokines such as interferon-γ (IFN-γ) [9,10]. Human NK cells have traditionally been classified into CD56 dim and CD56 bright subsets. The CD56 dim subset is generally regarded as the principal cytotoxic population, whereas the CD56 bright subset is considered more closely associated with cytokine production and immune regulation [11,12]. However, this functional dichotomy has not been fully understood and definitively confirmed. Wagner et al. showed that short-term interleukin-15 (IL-15) priming markedly enhanced the antitumor activity of CD56 bright NK cells, inducing degranulation, cytotoxicity, and production of IFN-γ and tumor necrosis factor (TNF) in response to tumor targets [13]. In multiple myeloma, NK cells have been reported to shift toward a dysfunctional CD56 bright cytotoxic phenotype with selective impairment of cytokine production, whereas the CD56 dim subset shows features of exhaustion, including impaired proliferation and increased PD-1 expression [14]. These findings indicate that CD56 bright NK cells can acquire a multifunctional antitumor effector phenotype under appropriate cytokine stimulation and that NK cell function cannot be inferred solely from the CD56-based subset classification. In addition, NK cells not only directly kill tumor cells but also regulate the broader tumor immune response. NK cells can promote dendritic cell maturation and support the induction of CD8+ T cell responses [15]. However, within the tumor microenvironment, their function is vulnerable to suppression by inhibitory cytokines, metabolic stress, and the increased expression of immune checkpoint molecules [10,15,16]. Thus, NK cells can support antitumor immunity beyond direct tumor-cell killing, but these functions are often suppressed within the tumor microenvironment.

OVCA cells are susceptible to NK cell-mediated cytotoxicity, and the abundance and functional status of NK cells have been associated with clinical outcomes [17,18,19,20]. NK cell dysfunction within the tumor microenvironment is not unique to OVCA. In other solid cancers, including non-small-cell lung cancer, breast cancer, colon cancer, and melanoma, tumor-infiltrating NK cells have been reported to acquire CD56 bright or CD16-low phenotypes, reduced cytotoxic capacity, tissue-adapted features, and checkpoint-mediated functional restraint [21,22]. A distinctive feature of OVCA is that many patients are diagnosed at an advanced stage with malignant ascites, peritoneal dissemination and omental metastasis, rather than with disease confined to the primary tubo-ovarian site [23,24]. This compartmentalized pattern is a distinctive feature of OVCA, because malignant ascites and their dissemination create highly immunosuppressive peritoneal niches that contain tumor cells, immune cells, stromal elements, soluble mediators, and metabolic factors [25,26,27]. Therefore, understanding NK cells in OVCA requires an integrated perspective that goes beyond the assessment of cytotoxic capacity. It is necessary to consider which compartment NK cells occupy, what phenotypic and functional states they exhibit, and how these states intersect with therapeutic resistance. Indeed, NK cell dysfunction in epithelial OVCA should not be viewed as a single, uniform abnormality. Rather, it should be understood as a spatially heterogeneous phenomenon shaped by compartment-specific soluble factors, metabolic conditions, and cell–cell interactions within peripheral blood, ascites, primary tumors, and peritoneal or metastatic lesions [19,20,28,29,30,31,32].

**Figure 1:**
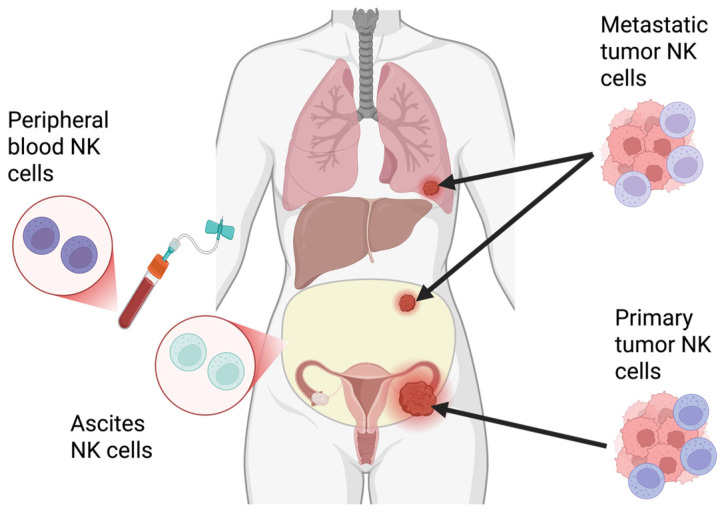
Compartment-specific NK cell states in epithelial ovarian cancer (OVCA).

In this review, we propose a compartment-based framework for understanding NK cell dysfunction in epithelial OVCA (Figure 1). We compare NK cell phenotypes and functional states across peripheral blood, ascites, primary tumors, and metastatic sites, and discuss how these differences shape distinct suppressive mechanisms and therapeutic vulnerabilities. A central premise is that no single compartment fully represents the overall NK cell landscape. Rather, peripheral blood, ascites, primary tumors, and metastatic lesions should be viewed as interconnected but functionally distinct immune environments, in which NK cell frequency, phenotype, signaling capacity, and effector function are differentially regulated. This framework may support biomarker development and inform the design of NK cell-targeted therapies tailored to the dominant disease compartment.

This schematic introduces the major compartments considered in this review, including peripheral blood, ascites, primary tumor, and metastatic lesions. These compartments provide distinct biological contexts in which natural killer (NK) cell phenotype and function may differ. The figure highlights the central premise of this review that NK cell states in epithelial OVCA should be evaluated across compartments rather than inferred from a single site.

## 2. Ascites-Associated NK Cell States in Ovarian Cancer

### 2.1. Ascites Contains Heterogeneous NK Cell Populations Distinct from Circulating NK Cells

Peripheral blood is the most accessible and less invasive compartment and provides a useful reference point for NK cell analysis. However, in OVCA, peripheral blood NK cells do not fully represent the phenotype or functional state of NK cells present in ascites [33,34,35]. In peripheral blood, NK cells are generally enriched for the CD16 + CD56 dim subset, whereas ovarian cancer-associated ascites contain a higher proportion of CD56 bright NK cells [33,34,35]. CD16- CD56 bright NK cells account for approximately 10% of peripheral blood NK cells but 40% of NK cells in peritoneal fluid from patients with epithelial OVCA [33]. This compartmental shift indicates that ascites NK cells cannot be interpreted simply as a mirror of circulating NK cells. The reduction or alteration of the CD16 + CD56 dim population is relevant because CD16/FcγRIIIa is the principal receptor mediating antibody-dependent cellular cytotoxicity (ADCC) [36]. CD16 does not signal independently but transmits ADCC signals through ITAM-containing adaptor chains, including FcRγ and CD3ζ, which recruit Syk- and ZAP70-related signaling pathways to promote NK-cell degranulation, cytokine production, and cytotoxicity [36,37]. Thus, the ascitic shift toward CD56bright NK cells may affect not only cytokine responsiveness but also natural cytotoxicity and ADCC-related killing.

**Figure 2:**
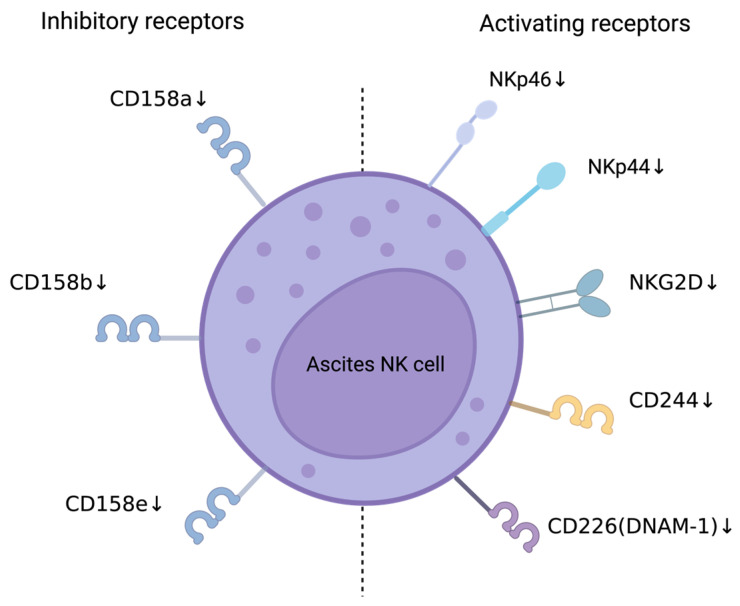
Phenotypic shift and reduced receptor expression in ascites-associated NK cells.

Phenotypic differences further support this distinction. Compared with peripheral blood NK cells, peritoneal fluid NK cells show reduced expression of several NK cell receptors, including NKp46, NKp44, NKG2D, CD244, CD226 (DNAM-1), CD158a, CD158b, and CD158e [33] (Figure 2). NKp46 and NKp44 are natural cytotoxicity receptors that contribute to tumor recognition, whereas NKG2D, CD244, and CD226 provide additional activating input [33,38,39,40]. In contrast, CD158a, CD158b, and CD158e represent KIR-related molecules and suggest that inhibitory tuning also differs between blood and ascites [33,41] (Figure 2). These receptor changes have mechanistic implications because NK cell recognition is regulated by the balance between activating receptors, inhibitory KIR-related receptors, and CD16-dependent effector function.

Compared with peripheral blood NK cells, ascites-associated NK cells are enriched for the CD56bright subset and show reduced expression of multiple NK cell receptors, including NKp46, NKp44, NKG2D, CD244, CD226/DNAM-1, and the KIR-related molecules CD158a, CD158b, and CD158e. Downward arrows indicate reduced expression.

Single-cell analysis of high-grade serous OVCA ascites further supports transcriptional heterogeneity among NK-like or cytotoxic lymphocyte populations. In this study, cells from treatment-naive high-grade serous ovarian cancer (HGSOC) ascites were classified into major cell types using transcriptomic marker genes, and NK cells were subsequently analyzed as a transcriptionally annotated subset [42]. Within this transcriptionally annotated NK cell compartment, distinct gene-expression patterns were observed, including metallothionein-related genes associated with metal handling and oxidative stress responses (MT2A, MT1G, and MT1X), interferon- or transcriptional-accosiated genes (IFI44L and SOX4), cytotoxic or T/NKT-associated genes (CD3D and GZMA), and inflammatory or myeloid-associated genes involved in monocyte/macrophage-associated inflammation and chemotaxis (LYZ, CD14, and CCL2) [42,43,44,45,46,47]. These findings suggest that ascites associated NK-like or cytotoxic lymphocyte populations are not uniform but include gene expression-defined stress response, and interferon-responsive, cytotoxic, and inflammatory or myeloid-associated states. Marked inter-patient and intra-patient heterogeneity was also observed, suggesting that NK cell states in ascites fluctuate according to the local microenvironment [42].

**Figure 3:**
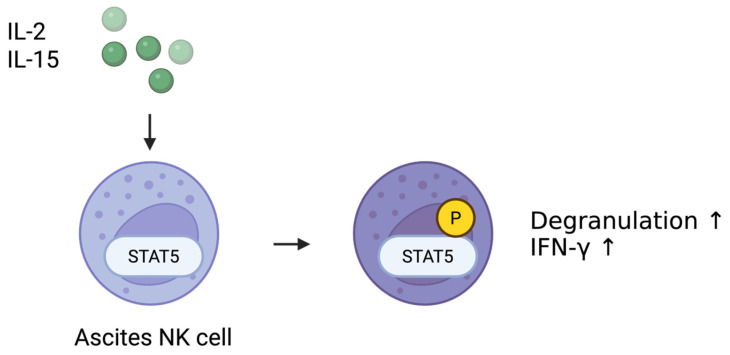
Cytokine responsiveness and functional recoverability of ascites-associated NK cells.

OVCA-associated ascites contains higher levels of interleukin-6 (IL-6) and IL-10 than peripheral blood, indicating that ascites provides an inflammatory but immunomodulatory cytokine milieu [34,48,49]. IL-2 and IL-15 are central cytokines for NK cell survival, proliferation, and functional activation, and their signaling capacity is therefore relevant to the potential reactivation of NK cells within OVCA-associated ascites [50,51]. In vitro stimulation with IL-2 or IL-15 induced STAT5 phosphorylation in NK cells from both blood and ascites, indicating preserved cytokine-inducible signaling capacity [34]. Within ascites, the expanded CD56 bright NK cell subset showed stronger IL-2-induced STAT5 phosphorylation than the CD56 dim subset, suggesting greater responsiveness to IL-2 [34] (Figure 3).

In patients with advanced OVCA, a higher proportion of intraperitoneal NK cells has been associated with better clinical outcomes, and ex vivo stimulation with IL-15 enhanced functional responses of ascites-derived NK cells against tumor target cells, including K562 and SKOV-3 cells [17] (Figure 3). Together, these observations suggest that ascites NK cells are not uniformly inert, but may include subsets that can be reactivated by appropriate cytokine stimulation.

IL-2 or IL-15 stimulation induces STAT5 phosphorylation in NK cells from ovarian cancer-associated ascites, indicating preserved cytokine-inducible signaling. IL-15 stimulation further enhances functional responses of ascites-derived NK cells against tumor target cells, supporting the concept that ascites NK cells include subsets with recoverable tumor-cell reactivity. Upward arrows indicate increased expression or functional activity.

Ovarian cancer ascites also contains tissue-resident-like cytotoxic lymphocyte populations. CD49a, also known as integrin α1 or ITGA1, pairs with CD29/integrin β1 and contributes to adhesion to extracellular matrix components such as collagen; in NK cells, CD49a is commonly used as a marker of tissue-resident or tissue-adapted states [52]. In high-grade serous ovarian cancer ascites, CD103 + CD49a+ NK cells and CD8+ T cells have been identified, and similar populations were also detected in primary tumor tissue [30]. Although these tissue-resident-like NK cells expressed high levels of NKG2A which forms an inhibitory CD94/NKG2A receptor complex that recognizes HLA-E, they maintained responsive to ovarian tumor cells in functional assays [30,41,53].

These findings indicate that NK cells in OVCA-associated ascites are not a single functional population but a heterogeneous compartment in which cytokine-responsive subsets coexist with subsets impaired by suppressive factors and altered receptor expression.

### 2.2. Soluble, Biochemical, and Lipid-Mediated Mechanisms Suppress Ascites NK Cell Function

**Figure 4:**
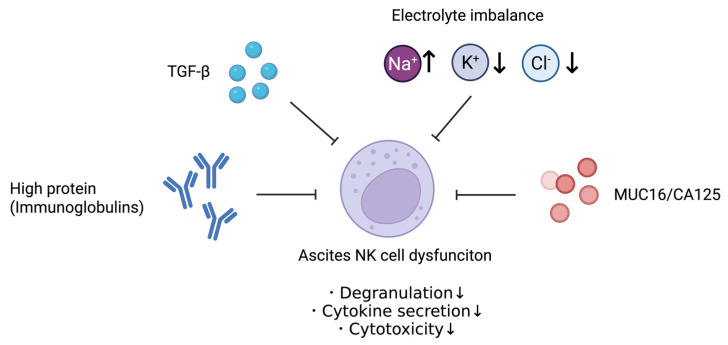
Soluble and biochemical suppressive factors in ovarian cancer-associated ascites.

In high-grade epithelial OVCA, increased peritoneal transforming growth factor-β1 (TGF-β1) has been associated with ascites-induced NK cell dysfunction and reduced survival [19]. Moreover, immune suppression in malignant ascites cannot be explained by cytokines alone. Aberrant electrolyte composition, including increased sodium and reduced chloride and potassium, and high concentrations of immunoglobulins and serum-derived proteins can impair NK cell effector function in malignant ascites [54,55] (Figure 4).

The mechanisms underlying these biochemical suppressive effects are partly defined. Electrolyte imbalance, particularly increased sodium together with reduced chloride and potassium, can suppress NK cell degranulation, tumor-cell killing, cytokine secretion, and calcium signaling, and may alter ion-channel expression and downstream signaling molecules involved in immune-cell activation [54] (Figure 4). In contrast, the mechanisms mediated by immunoglobulins and serum-derived proteins appear to be more heterogeneous. High protein concentrations can impair NK cell degranulation, conjugation to tumor cells, and intracellular calcium signaling, whereas ascites-derived immunoglobulins may competitively interfere with CD16-dependent ADCC and reduce NK–tumor cell conjugation [55] (Figure 4).

MUC16 (CA125) may suppress NK cell function not only as a soluble tumor-associated factor but also by interfering with NK cell recognition. High MUC16 expression inhibits immune synapse formation between NK cells and ovarian tumor cells [56]. This mechanism is consistent with the observation that MUC16/CA125 binds to a subset of CD16 + CD56dim NK cells in both peripheral blood and peritoneal fluid [33] and that OVCA ascites inhibits NK cell transcriptional activation partly through CA125 [57]. A previous study has shown that CA125 induced major downregulation of CD16 in a cell line experiment [58] (Figure 3). Therefore, MUC16/CA125 may preferentially compromise the CD16 + CD56dim population that is relevant to cytotoxicity and ADCC, although direct evidence linking CA125 to impaired ADCC in OVCA ascites remains limited.

Ovarian cancer (OVCA)-associated ascites contains multiple soluble and biochemical factors that impair NK cell function. Increased transforming growth factor-β1 (TGF-β1), electrolyte imbalance, MUC16/CA125, immunoglobulins and serum-derived proteins can contribute to ascites-associated NK cell dysfunction. These suppressive effects are linked to reduced degranulation, cytokine secretion, and cytotoxicity. Upward arrows indicate increased concentration, whereas downward arrows indicate reduced concentration or functional activity.

High concentrations of the soluble NKG2D ligands sMICA and sULBP2 in ovarian cancer-associated ascites have been associated with poor clinical outcome [59]. Although ascites inhibited normal NK cell activation, this effect was not associated with reduced NKG2D expression, contrary to the conventional model of soluble NKG2D ligand-mediated suppression [59]. The role of soluble NKG2D ligands in OVCA ascites is more complex than simple NKG2D downregulation and may reflect broader immune remodeling, including reduced effector memory T-cell populations, increased protumorigenic macrophages, and NKG2D ligand-independent suppression of NK cell activity [59].

**Figure 5:**
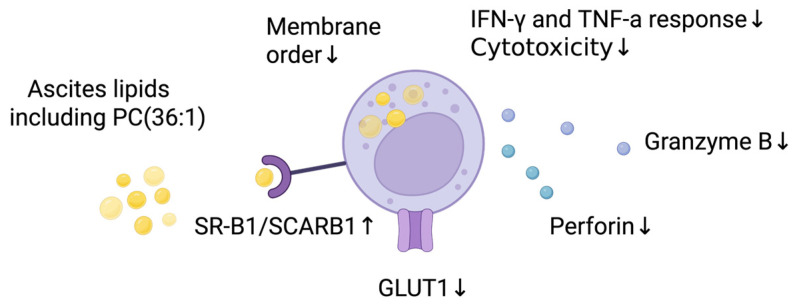
Lipid-mediated suppression of ascites-associated NK cell function in ovarian cancer.

NK cell antitumor activity is sensitive to lipid-driven metabolic stress with obesity-associated lipid accumulation imparing NK cell metabolic reprogramming and tumor-cell killing [60] A central mechanism of ascites-associated NK cell dysfunction is lipid-driven metabolic impairment [29]. Metabolomic and lipidomic analyses of samples from patients with HGSOC have shown that ascites is nutrient-rich but immunosuppressive for NK cells, T cells, and innate T cells, with uptake of polar lipids emerging as a major driver of this effect [29]. Ascites exposure markedly alters the lipid composition of NK cells, and phosphatidylcholine PC(36:1) has been identified as a representative immunosuppressive lipid [29]. Uptake of PC(36:1) disrupts intracellular lipid handling, reduces membrane order, and impairs immune synapse formation and polarization, which are required for cytotoxic activity [29] (Figure 5).

Importantly, lipid depletion from ascites increased expression of the glucose transporter receptor GLUT1 and restored NK cell effector functions, including granzyme B expression, IFN-γ and TNF-α production, and tumor-cell killing capacity [29]. Suggesting that lipids are not merely background components of ascites but active mediators of its immunosuppressive function. This lipid-dependent dysfunction involves specific lipid-transport pathways. Ascites NK cells show increased expression of several lipid-uptake molecules, including SCARB1, CD36, FABP4, and FABP5, with SCARB1 being the most strongly induced [29] (Figure 5). SCARB1 encodes scavenger receptor class B type 1 (SR-B1), a receptor involved in the uptake of lipids including phosphatidylcholine. Inhibition of SR-B1 restored granzyme B and perforin expression and improved NK cell cytotoxicity even in the presence of malignant ascites [29]. Thus, lipid uptake through SR-B1 provides a mechanistic link between the biochemical composition of ascites and impaired NK cell effector function.

Ovarian cancer (OVCA)-associated ascites contains immunosuppressive polar lipids, including phosphatidylcholine PC(36:1), that are taken up by NK cells through lipid-transport pathways involving SR-B1/SCARB1. Lipid uptake disrupts intracellular lipid handling and reduces membrane order, leading to impaired NK cell effector function. Lipid depletion or SR-B1 inhibition can restore GLUT1 expression, granzyme B and perforin expression, IFN-γ and TNF-α production, and NK cell cytotoxic responses against tumor cells. Upward arrow indicates increased expression, whereas downward arrows indicate reduced expression or functional activity.

## 3. Primary Tumor-Infiltrating NK Cells Are Locally Adapted and Functionally Constrained

**Figure 6:**
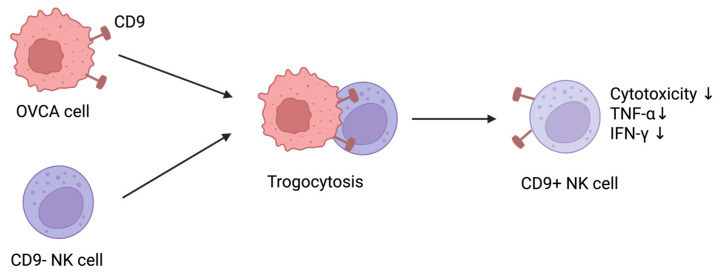
CD9-mediated suppression of NK-92 cell function after tumor-cell contact.

NK cells infiltrating primary ovarian tumors appear to be reshaped within the tumor tissue into locally adapted phenotypic states. In primary high-grade serous tubo-ovarian carcinoma, a decidual-like NK cell subset characterized by CD56 + CD9 + CXCR3 + KIR + CD3- CD16-has been identified. This subset correlates with both tumor cell and epithelial–mesenchymal transition-like cells abundance [32]. In tumor cell co-culture models, NK-92 cells acquired tumor-derived CD9 by trogocytosis. Compared with CD9^−^ NK-92 cells, CD9^+^ NK-92 cells showed lower frequencies and expression levels of TNF-α and IFN-γ after stimulation, together with impaired cytotoxicity [32] (Figure 6).

NK-92 cells acquire tumor-derived CD9 through trogocytosis during co-culture with OVCA cells. CD9^+^ NK-92 cells exhibit reduced TNF-α and IFN-γ production and impaired cytotoxicity, indicating that CD9 transfer can dampen NK cell antitumor function. Downward arrows indicate reduced expression or functional activity.

CXCR3 may facilitate recruitment or localization of NK cells within inflamed tumor tissue through the CXCL9/CXCL10/CXCL11 axis [61], whereas loss or reduction in CD16 may reduce the capacity of tumor-infiltrating NK cells to mediate antibody-dependent cellular cytotoxicity (ADCC) [36]. In parallel, KIR expression indicates that inhibitory receptor pathways may participate in local NK cell tuning [41,62]. This inhibitory pathway may recruit SHP-family phosphatases such as SHP-1 and thereby dampen proximal activation signaling. Additionally, SHP-2 has also been reported to negatively regulate NK cell activation in response to tumor target cells [41,62,63]. Thus, tumor-induced acquisition of a CD56 + CD9 + CXCR3 + KIR + CD3- CD16- phenotype should be interpreted as a combined state in which tumor-localizing NK cells shift away from CD16-dependent cytotoxicity and acquire inhibitory or tissue-adapted features [32].

Compared with peripheral blood NK cells, NK cells in primary ovarian tumor tissue show reduced frequencies and lower expression of the activating receptors NKp46 and DNAM-1, together with reduced CD57 expression and increased PD-1 expression [64]. DNAM-1, also known as CD226, is an adhesion and co-activating receptor expressed on NK cells and cytotoxic T cells. Its ligands, PVR/CD155 and Nectin-2/CD112, promote NK cell recognition of target cells and enhance NK cell-mediated cytotoxicity [65]. Expression of PVR, the ligand for DNAM-1, was significantly increased in tumor tissues, and in vitro experiments demonstrated that DNAM-1 contributed to NK cell-mediated lysis of primary tumour samples [64]. However, analysis of the relationship between NK cell phenotype and clinical outcomes showed that higher DNAM-1 expression on both tissue-localized and peripheral NK cells was associated with poorer survival [64]. This apparently paradoxical finding suggests that DNAM-1 expression may reflect persistent tumor-recognition pressure rather than effective antitumor immunity. Therefore, primary tumor-infiltrating NK cells appear to retain elements of DNAM-1-dependent tumor recognition, but their effector output is constrained by inhibitory checkpoints and the suppressive tumor microenvironment.

Integrated analyses of human HGSOC specimens and murine HGSOC models identified an NKG2A-enriched NK cell state with reduced cytotoxic features. In human samples, single-cell and spatial analyses showed enrichment of KLRC1/NKG2A-expressing NK cells, their localization within HLA-E-rich tumor areas, and an association with reduced granzyme B expression and poorer survival [53]. In murine HGSOC models, NKG2A blockade shifted NK cells toward an NKp46^+^IFN-γ^+^ phenotype and enhanced CD8+ T-cell responses, particularly when combined with PD-1 blockade [53]. These findings suggest that NKG2A blockade does not merely enhance NK cell cytotoxicity, but also reshapes the intratumoral CD8+ T-cell compartment toward an ICI-responsive progenitor-like exhausted state [53]. In addition, the increased frequency of PD-1 + TCF1/TCF7 + CD8+ T cells and the concomitant reduction in PD-1 + TIM3 + CD8+ T cells after NKG2A blockade suggest remodeling of the intratumoral CD8+T-cell compartment toward a less terminally exhausted, ICI-responsive state, providing a mechanistic rationale for combined NKG2A and PD-1 blockade [53].

**Figure 7:**
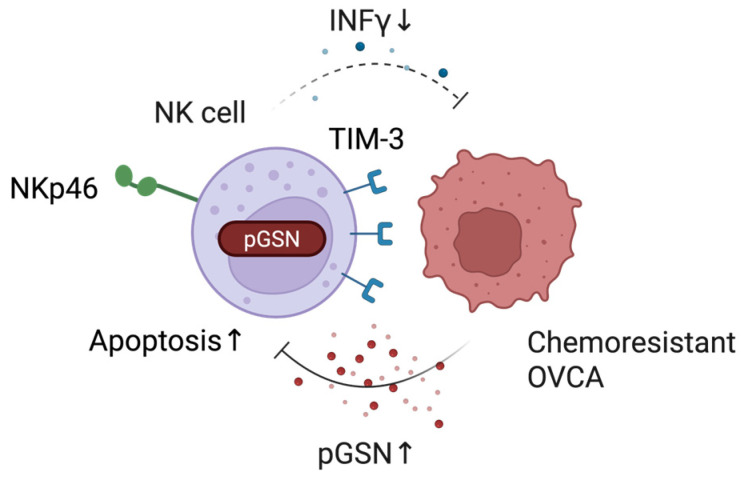
pGSN-mediated suppression of activated NK cells in chemoresistant ovarian cancer.

Soluble tumor-derived factors may further reinforce NK cell dysfunction in primary lesions. Plasma gelsolin (pGSN) is an actin-binding protein with oncogenic and immune suppressive properties which has been implicated in OVCA [66,67,68]. In analyses of primary surgical specimens from epithelial OVCA, infiltration by activated NK cells was associated with a survival advantage, whereas high pGSN expression attenuated this benefit [28]. Chemoresistant OVCA cells secrete higher levels of exosomal pGSN, which induces apoptosis of CD8+T cells and M1 macrophages, reduces IFN-γ secretion, increases glutathione production in OVCA cells, thereby conferring cisplatin resistance to OVCA cells [67]. Similarly, pGSN induces NK cell apoptosis through direct contact between chemoresistance OVCA cells and NK cells. pGSN also increased TIM-3 expression on activated NK cells and reduced IFN-γ production by TIM-3 +-activated NK cells [28] (Figure 7).

Chemoresistant ovarian cancer cells with pGSN expression suppress activated NKp46+ NK cells through direct cell contact. This interaction increases NK cell apoptosis and TIM-3 expression and reduces IFN-γ production, contributing to impaired NK cell antitumor function. Upward arrow indicates increased expression or functional activity, whereas downward arrows indicate reduced expression.

These findings suggest that primary tumor-associated NK cell dysfunction may be sustained not only by inhibitory receptors such as PD-1 and NKG2A but also by a pGSN-TIM-3-related suppressive pathway.

Thus, primary tumor-infiltrating NK cells should be viewed as a locally restrained population in which residual tumor-recognition signals are counterbalanced by impaired cytotoxic maturation, reduced CD16-dependent effector function, inhibitory checkpoint signaling, and soluble tumor-derived suppression [28,32,53,64].

## 4. NK Cells in Metastatic Lesions Share Suppressive Features with Those in Primary Tumors

NK cells in OVCA metastatic lesions show suppressive and functionally constrained features. In matched flow-cytometric analyses, metastatic omental lesions contained fewer NK cells than peripheral blood and showed reduced expression of NKp46, DNAM-1, and CD57, together with increased PD-1 expression [64]. These findings suggest that NK cells within metastatic lesions are characterized by diminished activating receptor expression and increased checkpoint-associated restraint, rather than simply reflecting differences in NK cell abundance. Single-cell RNA-seq of metastatic omental tissue further supports this interpretation at the NK cell transcriptional level. After NK cells were identified and analyzed as a separate subset, metastatic omental NK cells showed lower cytotoxicity module scores based on GZMA, GZMB, GZMH, GZMM, GNLY, PRF1, and CTSW, together with higher exhaustion and inhibitory receptor module scores that included CD39, PDCD1, TIM-3, CTLA4, KLRC1, KLRD1, KLRB1, LAIR1, FAS, and TIGIT [64]. These findings suggest that NK cells in omental metastases are transcriptionally shifted toward exhaustion- and inhibition-associated states.

In high-grade serous ovarian carcinoma, PD-1 + NK cells infiltrate both primary tumors and metastatic lesions, but they are more frequently observed in metastatic sites [20]. Spatial analyses demonstrate proximity between PD-1 + NKG2A+ NK cells and PD-L1 + HLA-E+ tumor niches in metastatic lesions, suggesting that NK cell suppression in metastatic lesions is coordinated by local ligand expression as well as by inhibitory receptor expression [20].

Consistent with the NKG2A-enriched dysfunctional NK cell state observed in primary HGSOC, single-cell analyses of an omental metastatic gradient showed that overt HGSOC omental metastases were enriched for dysfunctional NK cell states [53]. These NK cells showed increased expression of checkpoint-associated transcripts, including KLRC1, HAVCR2, and TIGIT, together with reduced expression of cytotoxic molecules (GZMA, GZMB, GZMH, PRF1, and NKG7) and activating receptor-associated transcripts, including CD160 and NCR3 [53]. Thus, metastatic lesions appear to share the suppressive NK cell program observed in primary tumors, with further enrichment of this program along the omental metastatic gradient.

Together, these findings suggest that NK cells in OVCA metastatic lesions share the suppressive phenotype seen in primary tumors, with reduced activating receptor expression and increased inhibitory checkpoint signaling. These features may be reinforced within the metastatic niche, indicating that metastatic NK cell dysfunction represents a locally intensified form of the functional restraint already observed in primary tumors [20,53,64].

## 5. Intraperitoneal Delivery as a Route-Specific Strategy for NK Cell Therapy in Ovarian Cancer

The compartment-specific nature of NK cell dysfunction in OVCA has direct implications for NK cell therapy, because disease dominated by ascites and peritoneal dissemination may require route-specific delivery strategies rather than systemic administration alone. Early clinical experience with adoptive NK cell therapy in ovarian cancer mainly used systemic delivery. In a phase II study of allogeneic NK cell therapy for recurrent ovarian and breast cancer, intravenously infused donor NK cells were evaluated for tumor response and in vivo expansion [69]. Although this study supported the clinical feasibility of systemic NK cell infusion, it also highlighted the need to improve in vivo NK cell persistence and expansion [69].

More recent clinical development has therefore focused on intraperitoneal delivery, which is anatomically suited to ascites-dominant and peritoneal disease. In the INTRO-01 phase I trial, intraperitoneal infusion of the stem cell-derived NK cell product RNK001 in patients with recurrent epithelial OVCA was feasible and well tolerated, without severe treatment-related toxicity, and transient reductions in CA125 were observed in several patients [70]. In preclinical OVCA xenograft models, intraperitoneal administration of NK-92 cells, either alone or combined with intravenous administration, showed more favorable survival effects than intravenous administration alone [71]. Together, these findings suggest that NK cell therapy for OVCA should not be designed solely around systemic delivery. This route-specific strategy differs from many NK cell therapy approaches in other cancer types, where systemic infusion remains common, including early NK-92 studies in renal cell carcinoma and melanoma [72,73]. Instead, local delivery, including intraperitoneal administration, should be considered when the dominant disease compartment is the peritoneal cavity.

## 6. Future Research Directions

An important future direction is to clarify the origin of compartment-specific NK cell states in OVCA. It remains unclear whether NK cells in ascites, primary tumors, and metastatic lesions mainly reflect selective recruitment of circulating NK cells, local conversion after exposure to ascites- or tumor-derived factors, or expansion of tissue-resident-like NK cell populations. In addition, paired multi-compartment NK cell analyses should also define how NK cells interact with other immune populations across disease sites [9]. NK cell function is unlikely to be determined by NK-intrinsic changes alone, because NK cells can influence, and be influenced by, CD8+ T cells, dendritic cells, macrophages, Tregs, and myeloid-derived suppressive populations within the tumor microenvironment [9,74].

Longitudinal studies should define how NK cells in each compartment change after chemotherapy and other therapies. Reviews of neoadjuvant chemotherapy-induced immune remodeling in OVCA have focused mainly on T cells, tumor-infiltrating lymphocytes, and PD-1/PD-L1 expression, while NK cell-specific evidence remains insufficient [75]. Chemotherapy has been associated with partial remodeling of peripheral blood NK cell receptor profiles in HGSOC, including changes in NKp30, NKp46, NKG2C, and KIR2DL2/L3/S3, together with changes in selected NK cell ligands such as MICA, PVR, and B7-H6 [76]. Therefore, future studies should focus on blood, ascites, and tumor tissue longitudinally before treatment, during neoadjuvant chemotherapy, at interval surgery, and at recurrence to investigate NK cell abundance and functionality.

Antigen-directed engineered NK cell therapies provide another promising therapeutic direction. In OVCA models, chimeric antigen receptor (CAR) memory-like NK cells targeting the membrane-proximal domain of mesothelin have shown encouraging antitumor activity [77]. Claudin-6-targeted CAR-NK cells have also demonstrated antitumor efficacy in both in vitro and in vivo OVCA models [78]. In the latter setting, combination with anti-PD-L1 antibody further enhanced antitumor activity, supporting a strategy that pairs strengthened tumor recognition with release from immune suppression [78]. Thus, NK cell therapy in OVCA is evolving toward CAR-NK approaches [77,78]. Future studies should clarify whether CAR-NK cells, like non-engineered NK cell therapies, achieve better tumor access and antitumor activity through intraperitoneal rather than systemic intravenous delivery in ascites-dominant or peritoneal disease [69,70,71,72,73,79].

## 7. Conclusions

NK cell dysfunction in epithelial OVCA should be understood as a compartment-dependent process shaped by distinct local disease environments. Across ascites, primary tumors, and metastatic lesions, NK cells are exposed to different combinations of soluble suppressive factors, tumor-associated ligands, biochemical and metabolic stress, stromal interactions, and inhibitory checkpoint signals. Thus, NK cell dysfunction in OVCA represents a spectrum of locally shaped functional states rather than a single uniform abnormality.

This compartmental perspective also has therapeutic implications. NK cell-directed strategies in OVCA should consider not only NK cell abundance, but also the dominant suppressive mechanisms and reversibility of NK cell dysfunction within each disease compartment. This framework may guide rational combinations of cytokine stimulation, checkpoint blockade, metabolic targeting, and adoptive or engineered NK cell therapy tailored to ascites, primary tumors, and metastatic lesions.

## Data Availability

No new data were created or analyzed in this study. Data sharing is not applicable to this article.

## Abbreviations

The following abbreviations are used in this manuscript:

B7-H6	B7 homolog 6
CA125	cancer antigen 125
CAR	chimeric antigen receptor
CD	cluster of differentiation
CXCL	X-C motif chemokine ligand
DNAM-1	DNAX accessory molecule-1
FABP	fatty acid-binding protein
GLUT1	glucose transporter 1
HGSOC	high-grade serous ovarian cancer
HLA-E	human leukocyte antigen-E
IFN-γ	interferon-γ
IL	interleukin
KIR	killer-cell immunoglobulin-like receptor
MCP-1	monocyte chemoattractant protein-1
MICA	MHC class I polypeptide-related sequence A
MUC16	mucin 16
NK	natural killer
NKT	natural killer T
OVCA	ovarian cancer
PC(36:1)	phosphatidylcholine 36:1
PD-1	programmed cell death protein 1
PD-L1	programmed death-ligand 1
PD-L2	programmed death-ligand 2
pGSN	plasma gelsolin
PVR	poliovirus receptor
SCARB1	scavenger receptor class B member 1
SHP-1	Src homology region 2 domain-containing phosphatase-1
SHP-2	Src homology region 2 domain-containing phosphatase-2
SR-B1	scavenger receptor class B type 1
STAT5	signal transducer and activator of transcription 5
TGF-β1	transforming growth factor-β1
TIM-3	T-cell immunoglobulin and mucin-domain containing-3
TNF	tumor necrosis factor
ZAP70	zeta-chain-associated protein kinase 70
